# Sarsasapogenin regulates the immune microenvironment through MAPK/NF-kB signaling pathway and promotes functional recovery after spinal cord injury

**DOI:** 10.1016/j.heliyon.2024.e25145

**Published:** 2024-01-26

**Authors:** Bing Fang, Liyue Wang, Song Liu, Mi Zhou, Hongpeng Ma, Nianwei Chang, Guangzhi Ning

**Affiliations:** a International Science and Technology Cooperation Base of Spinal Cord Injury, Tianjin Key Laboratory of Spine and Spinal Cord Injury, Department of Othopaedics, Tianjin Medical University General Hospital, Tianjin, China; b Department of Othopaedics, Affiliated Hospital of Qingdao Binhai University, Qingdao, China; c Tianjin University of Traditional Chinese Medicine, Tianjin, China

**Keywords:** Spinal cord injury, Sarsasapogenin, Network pharmacology, Immune microenvironment, MAPK/NF-kB signaling pathways

## Abstract

Spinal cord injury (SCI) occurs as a result of traumatic events that damage the spinal cord, leading to motor, sensory, or autonomic function impairment. Sarsasapogenin (SA), a natural steroidal compound, has been reported to have various pharmacological applications, including the treatment of inflammation, diabetic nephropathy, and neuroprotection. However, the therapeutic efficacy and underlying mechanisms of SA in the context of SCI are still unclear. This research aimed to investigate the therapeutic effects and mechanisms of SA against SCI by integrating network pharmacology analysis and experimental verification. Network pharmacology results suggested that SA may effectively treat SCI by targeting key targets such as TNF, RELA, JUN, MAPK14, and MAPK8. The underlying mechanism of this treatment may involve the MAPK (JNK) signaling pathway and inflammation-related signaling pathways such as TNF and Toll-like receptor signaling pathways. These findings highlight the therapeutic potential of SA in SCI treatment and provide valuable insights into its molecular mechanisms of action. *In vivo* experiments confirmed the reparative effect of SA on SCI in rats and suggested that SA could repair SCI by modulating the immune microenvironment. *In vitro* experiments further investigated how SA regulates the immune microenvironment by inhibiting the MAPK/NF-kB pathways. Overall, this study successfully utilized a combination of network pharmacology and experimental verification to establish that SA can regulate the immune microenvironment via the MAPK/NF-kB signaling pathway, ultimately facilitating functional recovery from SCI. Furthermore, these findings emphasize the potential of natural compounds from traditional Chinese medicine as a viable therapy for SCI treatment.

## Abbreviation

SCI: Spinal cord injury
SA: Sarsasapogenin
MAPK: Mitogen activated protein kinase
NF-kB: Nuclear factor kappa-B
PPI: Protein-protein interaction
GO: Gene Ontology
KEGG: Kyoto Encyclopedia of Genes and Genomes
BPs: Biological processes
CCs: Cellular components
MFs: Molecular functions
BBB: Basso, Beattie, and Bresnahan
DMEM: Dulbecco's modified eagle medium
BCA: Bicinchoninic acid
PVDF: polyvinylidene fluoride
DC: Degree centrality
BC: Betweenness centrality
CC: Closeness centrality
LPS: Lipopolysaccharide
TNF: Tumor necrosis factor

## 1 Introduction

Spinal cord injury (SCI) is defined as damage to the spinal cord caused by traumatic events, resulting in temporary or permanent impairment of neurological function [1,2]. The worldwide incidence of SCI is approximately 50 per million people per year, and this number is increasing globally each year [3]. Following the initial primary damage, secondary injuries, such as inflammation, oxidative stress, and apoptosis can have even more serious consequences [4,5]. Among the various of secondary injury, changes in the immune microenvironment are the primary cause, leading to the expansion of the lesion [6]. Numerous studies have demonstrated that by mitigating the abnormal and sustained activation of the immune microenvironment, it is possible to decrease secondary damage and enhance neurological function recovery [[7], [8], [9]].

Sarsasapogenin (SA) is a naturally occurring steroidal compound found in Zhimu (*Rhizoma Anemarrhenae*), primarily in China, Japan, Korea and other Asian countries [10,11]. This compound has been extensively studied for its pharmacological applications and is commonly used to treat Alzheimer's disease, diabetic nephropathy, and memory impairment associated with aging [[12], [13], [14]].

As a significant regulator of the immune microenvironment, SA has been shown to effectively suppress inflammation by inhibiting the expression of pro-inflammatory M1 markers in central nervous system disease [[15], [16], [17]]. However, the therapeutic efficacy, bio-safety, and mechanisms of SA on SCI are still not well understood. The use of network pharmacology methods can improve the efficiency of drug target identification and provide new avenues for exploring drug-disease interaction mechanisms in clinical research [18,19]. Therefore, in our study, we employed network pharmacology to predict the treatment effect of SA on SCI and uncover its underlying mechanism. We identified 41 genes that were common to both SCI-related genes and SA target genes, and further conducted a protein-protein interaction (PPI) analysis using Cystoscope software. Additionally, we performed the Kyoto Encyclopedia of Genes and Genomes (KEGG) pathway and Gene Ontology (GO) annotation. Subsequently, a molecular docking study was carried out on the hub proteins and SA. Furthermore, we hypothesized that SA could potentially repair SCI by suppressing inflammation through the MAPK/NF-kB signaling pathways. To validate these predictions, we conducted experiments both in vivo and *in vitro*. Our findings, based on network pharmacology and experimental verification, present the first report on the therapeutic effect and underlying mechanism of SA in recovering from SCI.

## 2 Materials and methods

### 2.1 Acquisition of SA and SCI targets

To identify relevant target genes for SA, a compound with a simple chemical structure, we utilized the SwissTargetPrediction database to screen for targets with a probability greater than 1. Additionally, we conducted a review of relevant published literature to supplement the identified targets. The identified targets were subsequently normalized against the UniProt database. To further explore the potential therapeutic applications of SA for SCI, we conducted searches in databases such as GeneCard, DrugBank, TTD, DisGeNET, and OMIM using the term ‘Spinal cord injury’. The genes related to SCI that were collected from these databases were merged and rectified using the UniProt database [20]. Subsequently, we used R software (v.4.1) to intersect the disease target with the drug target, and finally, we created a Venn diagram to visualize the data.

### 2.2 Acquisition of PPI network and hub genes

A PPI network was constructed using the online database STRING [21], with an overall score threshold of >0.4. The CytoHubba software was then utilized to determine the relative weights of all genes and identify the hub genes within the PPI network. To uncover functional modules, we employed the Molecular Complex Detection (MCODE) algorithm in Cytoscape software (V3.8) [22].

### 2.3 KEGG and GO analysis

The GO system is widely used for classifying and describing the functions of gene products. Another valuable tool, the KEGG pathway, is a scientific database that helps analyze gene function systems and identify biologically enriched pathways. To investigate the molecular functions (MFs), biological processes (BPs), cellular components (CCs), and specific signaling pathways associated with SA anti-SCI, we employed the ClusterProfiler package in R software (v.4.1) with a significance threshold of *P* < 0.05 [23].

### 2.4 Molecular docking

The protein products of hub genes have their molecular structure derived from the protein database PDB. We obtained the molecular structure of the drug SA (CID:92095) from PubChem. To prepare for the docking assay, we processed the data using AutoDock Tool (V1.5.6) and obtained its PDBQT file format through PyMOL software [24].

### 2.5 Animals and drug administration

Female Wistar rats (8 weeks of age, weight: 190–210 g) were obtained from Beijing Vital River Experimental Animal Technology Co., Ltd. The rats were kept under standard conditions, including a temperature range of 20–25 °C, a humidity range of 40–60 %, and a 12-12-h light-dark cycle. They were provided with laboratory standard tap water and food. Ethical approval for all animal experiments described in the manuscript was granted by the Ethics Committee of 10.13039/501100010104Tianjin Medical University General Hospital. The specific approval number is (IRB2023-WZ-030). All procedures were performed following the guidelines set by the National Institutes of Health Guide for the Care and Use of Laboratory Animals, ensuring the highest standards of animal welfare. All experimental procedures were carried out following the ARRIVE guidelines (http://arriveguidelines.org/). The intervention doses of SA were determined based on previous studies [25,26]. Rats were treated with 5, 10, and 20 mg/kg of SA (Beijing Solarbio Science & Technology Co. Ltd, China) through intragastric administration once per day until the rats were sacrificed. The Sham group was administered with PBS.

### 2.6 SCI rat model preparation

All procedures were conducted under sterile conditions. Prior to surgery, the rats were anesthetized with inhaled isoflurane (R510-22, RWD, China), and the majority of their dorsal skin on the thoracic region was shaved. A 1.5 cm dorsal incision was made on the T10 vertebra, exposing the underlying vertebrae. A spinal cord dorsal laminectomy was then performed to expose the T10 vertebra, following the previously described method [27]. The contusion SCI model was created using the NYU Impactor (Model III, W.M.Keck, USA), as described in the literature. This involved using an impactor weighing 10 g and dropping it from a height of 25 mm. The wound was closed by suturing the muscle layers and the skin. In the Sham group, only a laminectomy was performed. After the surgery, the rats were given an intraperitoneal injection of 0.9 % saline solution to maintain their body fluids. Antibiotics were administered twice daily for the first three days post-injury. Manual urination was performed at least twice a day after the surgery to empty the bladder.

### 2.7 Basso beattie and bresnahan (BBB) locomotor scores

The locomotor function was evaluated using the Basso, Beattie, and Bresnahan (BBB) test [28]. The BBB test was performed one day prior to the injury, on the first day of the SCI, and then weekly thereafter. In this test, lower limb movement was assessed on a scale of zero to twenty-one. The hindlimb movement was visually observed in a plexiglass observation chamber for a 5-min period. The testing was conducted weekly for 8 weeks following the SCI, and all investigators were unaware of the experimental conditions.

### 2.8 CatWalk assessment gait analysis

Rat locomotion recovery was evaluated using the Cat Walk XT system by Noldus (version 10.6, Noldus, Netherlands). The assessment followed the instructions provided in the Cat Walk XT 10.6 Reference Manual and a previous study [29]. Briefly, rats that were 8 weeks post-injury voluntarily walked on a glass walkway illuminated by a fluorescent light in a dark environment. A camera positioned beneath the glass recorded their footprints. The Cat Walk XT software, in conjunction with the apparatus, calculated various statistics related to dimensions, time, distances, and relationships between footprints. Each animal performed three consecutive compliant runs at each time point.

### 2.9 Electrophysiological tests

In this study, we evaluated neural conduction in rats at 8 weeks post-injury using electrophysiological tests [30]. The rats' motor evoked potentials (MEP) were processed using an electrophysiological apparatus (YRKJ-G2008; Zhuhai Yiruikeji, China). The detection results were then used to assess the recovery of conductible function in rats.

### 2.10 Immunofluorescence staining

For immunofluorescence staining of spinal cord tissue, the spinal cord tissue was dehydrated in 30 % sucrose solution for 3 days, embedded in OCT, and cut into 10 μm thick sections in transverse section. Cryofixed spinal cord sections were permeabilized and blocked in TBST with 1 % BSA at ambient temperature for 1 h. The sections were then incubated overnight at 4 °C with the following antibodies: Anti-NeuN (1:300, Abcam, USA), Anti-CD68 (1:200, Abcam, USA), Anti-iNOS (1:200, Abcam, USA), and Anti-Arg1 (1:200, Abcam, USA). To visualize the sections, Cy3-labeled goat anti-mouse (1:500, Beyotime, China) and Alexa Fluor 488 goat anti-rabbit (1:500, Beyotime, China) were used as secondary antibodies. Nuclear detection was performed by applying Fluoroshield Medium containing DAPI (Abcam, USA). Finally, confocal microscopy was used to capture images of the sections.

### 2.11 Cell culture and drug treatments

BV2 cells were obtained from the Cell Bank of the Chinese Academy of Sciences and cultured in Dulbecco's modified Eagle's medium (DMEM, Gibico) supplemented with 10 % fetal bovine serum at 37 °C and 5 % CO_2_. To induce inflammation or polarization towards the M1 phenotype, lipopolysaccharide (LPS, Sigma, USA) and interferon-gamma (INF-gamma, Sigma, USA) were added to the medium at final concentrations of 100 and 20 ng/mL, respectively. Cell viability after treatment with SA was assessed using the CCK-8 assay. BV2 cells were seeded in 96-well plastic culture plates and incubated for 24 h before being treated with different concentrations of SA (0, 0.1, 1, 10, or 100 M) for an additional 24 h. The culture medium was removed and replaced with DMEM, followed by the addition of CCK-8 solution. After a 2-h incubation, the absorbance at 450 nm was measured using a microplate reader.

### 2.12 ELISA assays

For ELISA experiments, spinal cord tissue was collected to a specific length of 1 cm from the lesion epicenter. The tissue was then homogenized and digested using RIPA buffer with protease inhibitors to ensure efficient extraction of proteins of interest. The homogenate was centrifuged at 12,000 rpm for 15 min at 4 °C, and the supernatant was collected for subsequent ELISA analysis to quantify. Cytokines in cell supernatant and tissue homogenates were detected using ELISA kits (Jiangsu Enzyme Immune Industrial Co., Ltd., China), following the manufacturer's instructions.

### 2.13 Western blot

Cell proteins were extracted using RIPA buffer containing a mixture of protease and phosphatase inhibitors. The protein content of each sample was quantified using the bicinchoninic acid assay method with bovine serum albumin (Biosharp, China). Subsequently, an equal amount of protein was subjected to electrophoresis on a polyacrylamide gel and transferred to the PVDF membrane. The following antibodies were applied: anti-JNK (1:1000, Abcam, USA), anti-phospho-JNK (1:1000, Abcam, USA), anti-p65 (1:1000, Abcam, USA), anti-phospho-p65 (1:1000, Abcam, USA), and anti-GAPDH (1:1000, Abcam, USA). Finally, the chemiluminescence detection system (Immobilon Western, USA) was used to visualize the immunolabeled bands.

### 2.14 Cellular thermal shift assay (CETSA)

The CETSA experiment was conducted following the previously described protocol. After three freeze-thaw cycles, BV2 cells were collected and lysed in PBS containing protease inhibitors. The cell lysate was then diluted with buffer and divided into two parts. One part was treated with SA (100 mM) and the other part with PBS for 20 min at room temperature. The lysates from both aliquots were then divided into 50 μl volumes and heated at different temperatures (55, 60, 65, 70, 75, 80, and 85 °C) for 3 min. Subsequently, the samples were centrifuged and analyzed using a Western blot.

### 2.15 Flow Cytometry Analysis

BV2 cells were filtered through a 70 μm cell filter and incubated with Zombie NIR™ Dye (Biolegend, USA) for 15 min, followed by termination with Cell Staining Buffer. FITC anti-rat/human CD11b antibody (Biolegend, USA), APC anti-mouse F4/80 Antibody (Biolegend, USA), and PerCP/Cyanine5.5 anti-mouse CD86 Antibody (Biolegend, USA) were then incubated for 40 min. The cells were permeabilized with Cyto-Fast™ Fix/Perm buffer for 20 min. Subsequently, the cells were incubated with PE anti-mouse CD206 (MMR) antibody (Biolegend, USA) in darkness at room temperature for 20 min and terminated with Cyto-Fast™ Perm/Wash buffer. Samples were collected using BD LSRFortessa flow cytometry (BD Bioscience, USA) and data analysis was performed using FlowJo.

### 2.16 Statistical analysis

The statistical analysis was performed using GraphPad Prism 8.0 (San Diego, CA, USA). All data were presented as mean ± SEM. *P* < 0.05 was considered statistically significant. The two independent groups were compared using a two-sample *t*-test. Multiple comparisons were performed using one-way ANOVA analysis or two-way ANOVA with Dunnett or Tukey post hoc tests, Bonferroni-corrected for multiple comparisons.

## 3 Results

### 3.1 The intersection target between SCI and SA and PPI interaction network

**Fig. 1:**
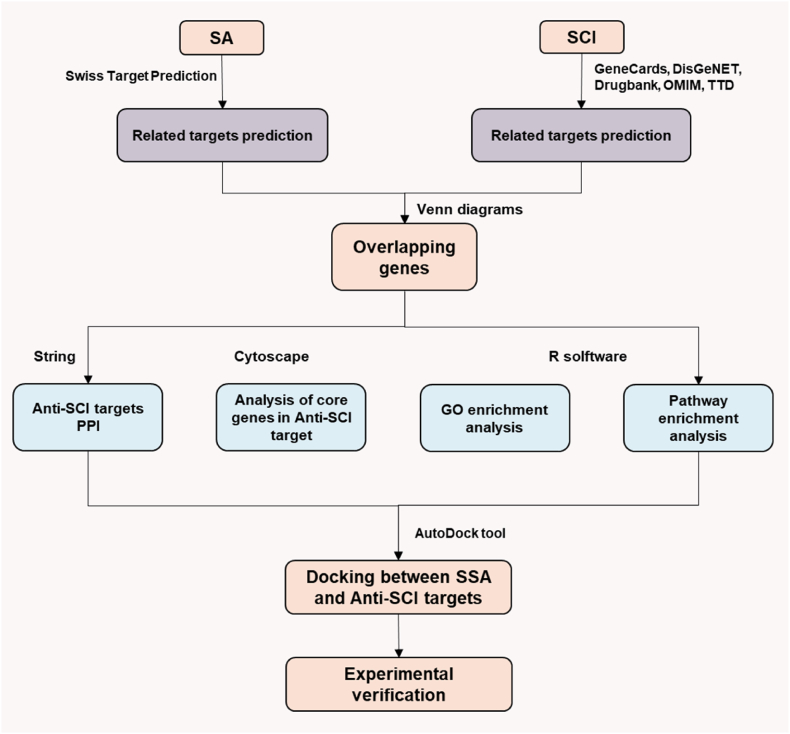
Workflow for the present study.

**Fig. 2:**
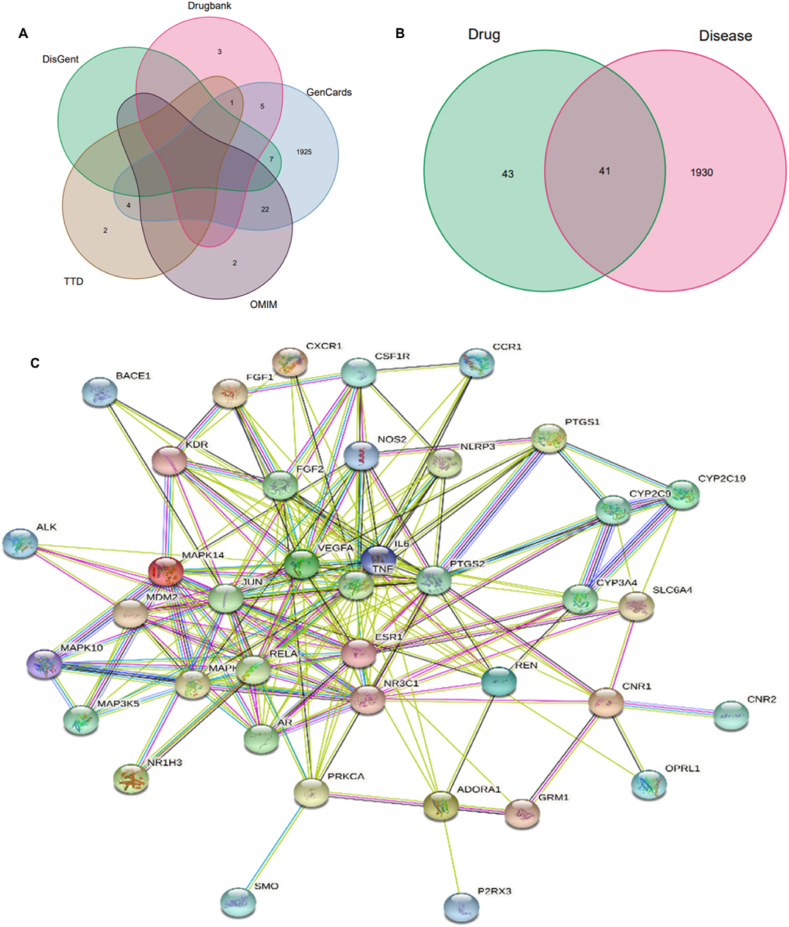
The drug-target-disease network and PPI network were constructed using the STRING database. **(A)** The collection of targets related to SCI. **(B)** Venn diagram depicting the overlap between targets related to SCI and targets related to SA. **(C)** The PPI network constructed using the STRING database.

Fig. 1 provides a schematic representation of the study's comprehensive experimental workflow. The SwissTargetPrediction database and literature were utilized for target prediction and supplementation for SA, resulting in the identification of 84 target genes associated with SA (Supplementary Table S1). In parallel, an extensive search across GeneCards, OMIM, TTD, DrugBank, and DisGeNET yielded 1971 target genes implicated in SCI (Supplementary Table S2). Among these data sets, we found an intersection of 41 shared genes between the SA-associated and SCI-related targets (Supplementary Table S3; Fig. 2A and B). This overlap facilitated the construction of a PPI network utilizing STRING analysis, comprising 40 nodes and 400 connecting edges (Fig. 2C).

**Fig. 3:**
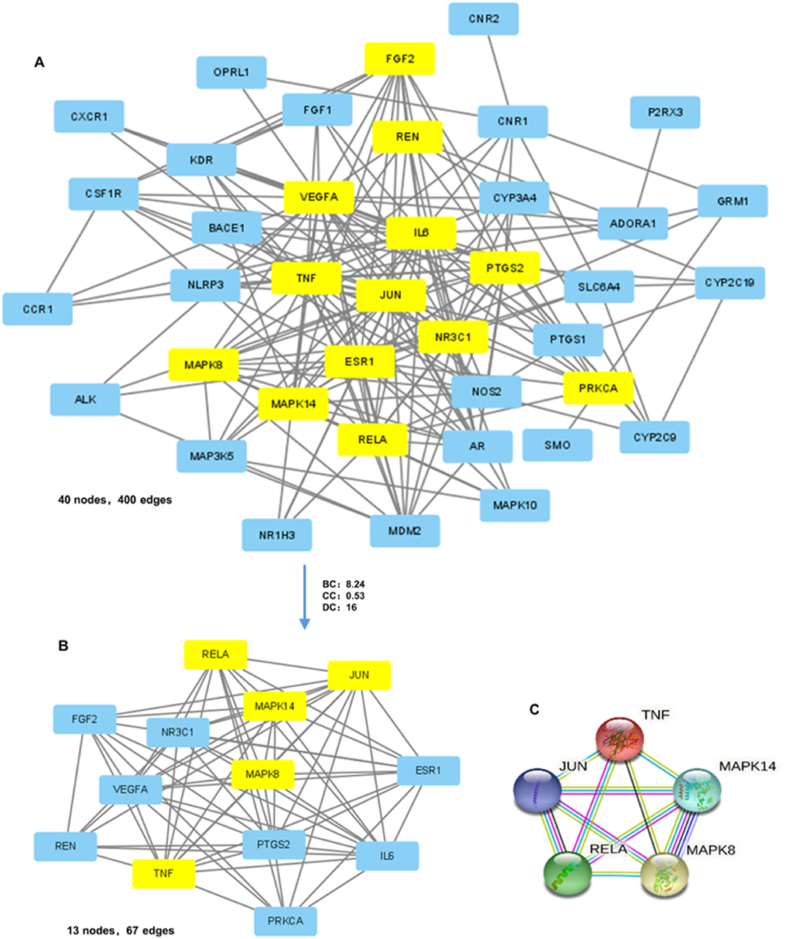
Construction of PPI network and acquisition of Hub Genes. **(A**–**B)** PPI network of predicted targets of SA against SCI and the significant proteins of the PPI network were extracted. **(C)** Screening 5 key proteins of SA in treating SCI.

The PPI network reflects a complex web of interactions among common genes. A meticulous evaluation of network topology identified nodes significantly enriched for drug targets, predicated on degree centrality (DC) values that were at least twice the median DC within the network. A median DC score of 16 served as the demarcation threshold for significant nodes, aiding in the distillation of a high-confidence subnetwork. This network filtration highlighted hub genes with betweenness centrality (BC) and closeness centrality (CC) indices surpassing the median network values (BC = 8.24, CC = 0.53), which served as the foundation for a core interaction network (Fig. 3A and B). Our integrative analyses elucidated a list of pivotal genes central to the therapeutic impact of SA on SCI. Notably, key inflammation and signaling mediators such as TNF, RELA, JUN, MAPK14, and MAPK8 emerged as potential therapeutic targets, sketching out a framework for potential intervention in SCI pathogenesis (Fig. 3C). These findings offer a molecular springboard for future investigations into tailored therapeutics for SCI informed by SA bioactivity.

### 3.2 Enrichment analysis of intersection targets of SCI and SA

**Fig. 4:**
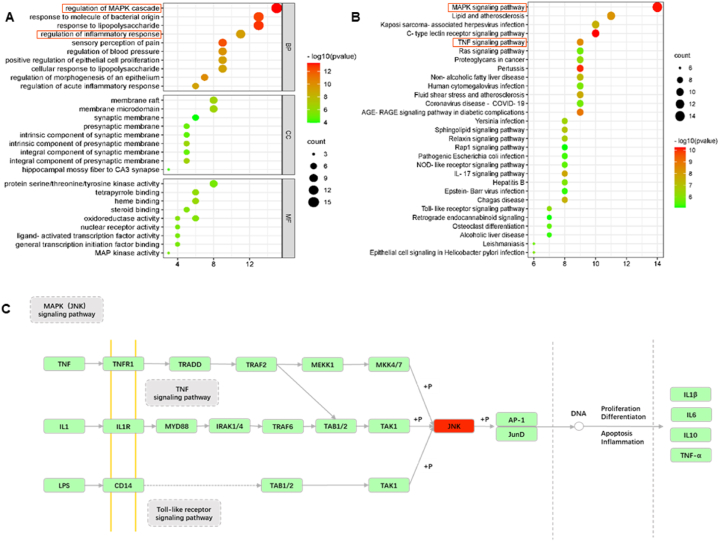
GO and KEGG enrichment analysis. **(A)** The top 10 of GO enrichment analysis in BPs, CCs, and MFs. **(B)** The top 30 signaling pathways were analyzed by KEGG. **(C)** The MAPK (JNK) signaling pathway and inflammation-related pathway (TNF and Toll-like receptor signaling pathway) were described in detail.

Through GO enrichment analysis, the therapeutic targets of SA against SCI were investigated across the frameworks of BPs, MFs, and CCs (Fig. 4A). The GO categorization exposed a concentration of targets engaged in cellular responses to chemical stimuli of bacterial provenance, including reactivity to lipopolysaccharides (LPS) and components related to inflammation, along with associations with membrane microdomains and rafts. Building on these insights, we carried out a KEGG pathway analysis to delineate the signaling cascades implicated by these potential targets. The findings revealed the top 30 pathways significantly enriched in the context of SCI, with pathways such as MAPK, TNF, and those mediating apoptosis among those most prominently featured (Fig. 4B). Notably, within this array, it was discerned that the MAPK (JNK) pathway and pathways guiding inflammation-related signals, inclusive of TNF and Toll-like receptor signaling, bore the most substantial linkages to SCI. The conjectured targets alongside their respective implicated signaling pathways are depicted within Fig. 4C. This repertoire of biological pathways offers a rich cartographic representation of the molecular interplay underlying SCI and sets the stage for targeted therapeutic intervention influenced by SA's bioactive spectrum.

### 3.3 Molecular docking verification of key targets

**Fig. 5:**
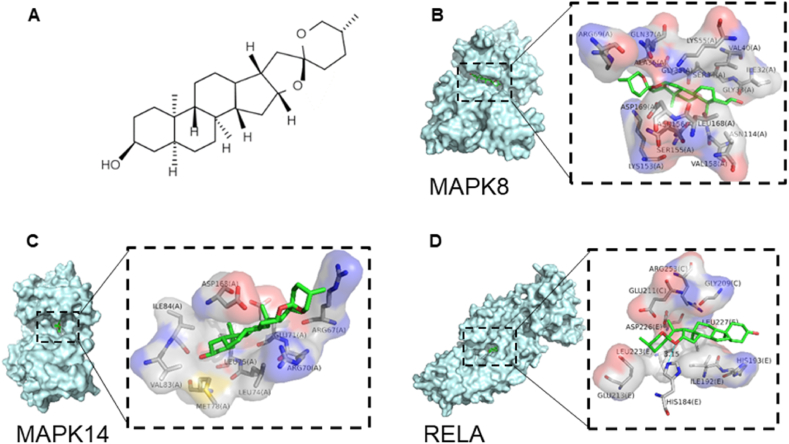
Molecular docking between SA and MAPK8, MAPK14, and RELA. **(A)** The chemically structural formula of SA. **(B)** SA and MAPK8. **(C)** SA and MAPK14. **(D)** SA and RELA.

In our compound-target interaction analysis, the salient chemicals (Fig. 5A) within the network were subjected to molecular docking trials against key protein targets, specifically MAPK8, MAPK14, and RELA (Fig. 5B–D). This in silico approach yielded binding affinities of −7.6 kcal/mol for SA with MAPK8 (JNK), −6.8 kcal/mol against MAPK14 (P38), and −5.6 kcal/mol with RELA (P65), respectively. Drawing upon the findings of Liu, Y. et al., we interpret these results to mean that the strength of a compound's binding affinity to a protein is inversely proportional to the AutoDock Vina scoring, the more negative the score, the more robust the interaction between the compound and its target. The docking outcomes, marked by a pronounced affinity for MAPK8 (JNK), suggest a substantial binding propensity of SA toward these integral signaling proteins [31], underpinning a molecular basis for the therapeutic action of SA in the context of SCI.

### 3.4 SA promoted the recovery of motor function after SCI in rats

**Fig. 6:**
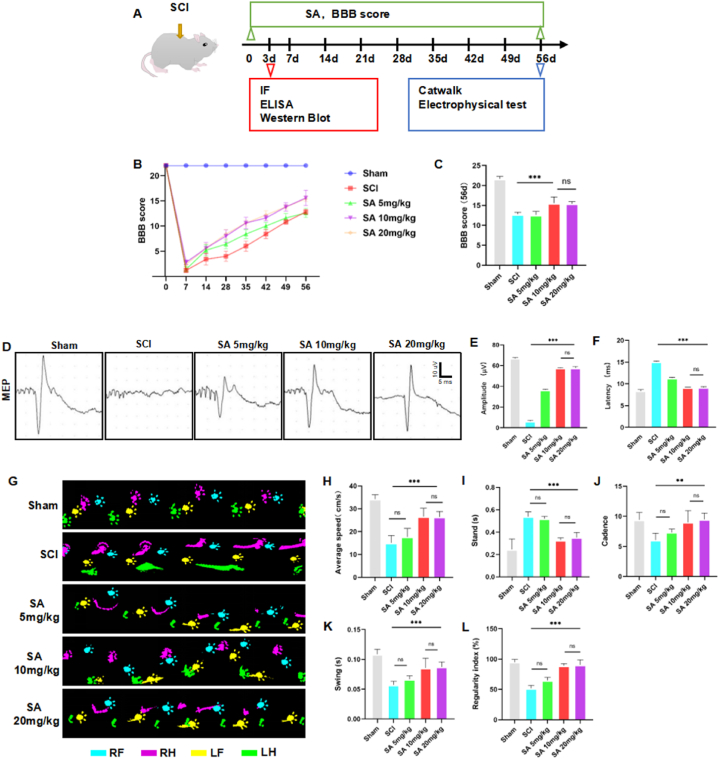
SA improved the recovery of motor functional outcomes after SCI. **(A)** Illustration describing the experiment design for long-term experiments and motor function recovery. **(B)** The degree of recovery was assessed for 8 weeks after SCI by BBB score (data are shown as mean ± SEM, two-way ANOVA with Tukey's post hoc test, *P < 0.05, **P < 0.01, ***P < 0.001 vs. the SCI group, n = 5). **(C)** Comparison of BBB scores of each group at 8 weeks after SCI (data are shown as mean ± SEM, one-way ANOVA with Tukey's post hoc test, *P < 0.05, **P < 0.01, ***P < 0.001 vs. the SCI group, n = 5). **(D)** Representative MEP waveform of nerve electrophysiology examination of rats in each group at 8 weeks after SCI. (**E, F**) Quantification of the latency and amplitude of MEP in each group at 8 weeks after SCI (data are shown as mean ± SEM, one-way ANOVA with Tukey's post hoc test, *P < 0.05, **P < 0.01, ***P < 0.001 vs. the SCI group, n = 6). **(G)** Representative footprints of animals 8 weeks after SCI. **(H**–**L)** Statistical analysis of average speed, stand, cadence, swing, and regular index of both hindlimbs (data are shown as mean ± SEM, one-way ANOVA with Tukey's post hoc test, *P < 0.05, **P < 0.01, ***P < 0.001 vs. the SCI group, n = 5, LF: left forelimb, RF: right forelimb, LH: left hindlimb, RH: right hindlimb).

To evaluate the therapeutic influence of SA on SCI, we employed a rat contusion model as depicted in Fig. 6A. Variable dosages of 5 mg/kg, 10 mg/kg, and 20 mg/kg SA were administered to delineate the optimal concentration for pharmacological mitigation. Post-treatment, the BBB locomotor rating scale was utilized to gauge functional recovery on a weekly timetable among the differentiated cohorts (Fig. 6B). Motor function restoration exhibited notable enhancements in rat assemblies treated with both 10 mg/kg and 20 mg/kg SA (Fig. 6C).

Further investigations using electrophysiological assessment at the 8-week juncture post-injury presented an augmented MEP amplitude in the 10 mg/kg and 20 mg/kg SA-treated groups (Fig. 6D and E). We also noted a reduction in latency in these same groups when compared to the untreated SCI cohort (Fig. 6F), though there were no discernible discrepancies between the 10 mg/kg and 20 mg/kg treatments. Nonetheless, SA administration was affirmed as an effective modality for fostering locomotor recuperation in the rat SCI model.

To substantiate these observations, gait analysis using the CatWalk XT system was conducted 8 weeks subsequent to the SCI event (Fig. 6G-L). The outcomes highlighted substantial gains in various gait metrics such as stand time, cadence, regularity index, average speed, and swing phase in the hindlimbs across recipients of both 10 mg/kg and 20 mg/kg SA dosages. The data indicates ameliorated strength and coordination in the posterior extremities following SA treatment in the SCI-afflicted rats. In the collective ambit of these findings, it is discerned that SA treatment at the dosages of 10 mg/kg and 20 mg/kg distinctly enhances motor function and hindlimb coordination post-SCI. From these results, the 10 mg/kg SA dosage was selected for subsequent investigative endeavors. This choice is justified by the inferred parity in efficacy to the higher dosage of 20 mg/kg, yet the 10 mg/kg dose showcased superior differential benefits relative to the lower 5 mg/kg dosage.

### 3.5 SA treatment inhibits inflammatory response in SCI rats and increases neuronal survival

**Fig. 7:**
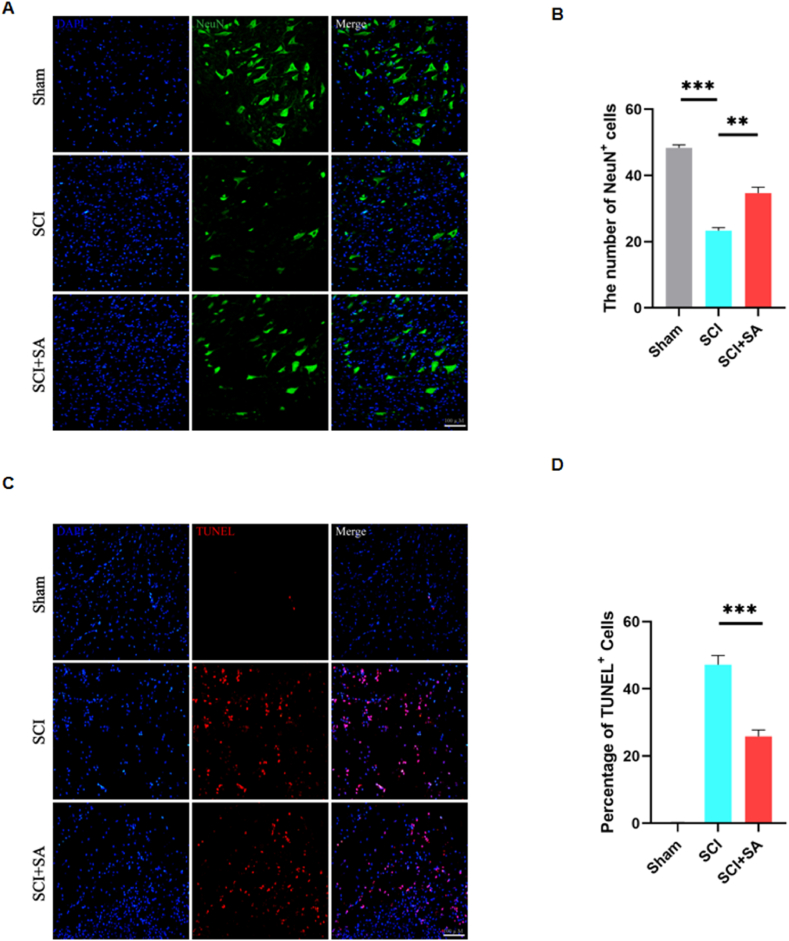
SA increased the number of surviving neurons and reduce apoptosis. **(A)** IF staining of NeuN in spinal cord sections at day 3 post-SCI. **(B)** NeuN-positive cells in the ventral horn of the spinal cord were quantified (n = 3). **(C)** IF staining of TUNEL in spinal cord sections at day 3 post-SCI. **(D)** TUNEL-positive cells in the ventral horn of the spinal cord were quantified (n = 3). Data are shown as mean ± SEM, one-way ANOVA with Tukey's post hoc test, *P < 0.05, **P < 0.01, ***P < 0.001.

**Fig. 8:**
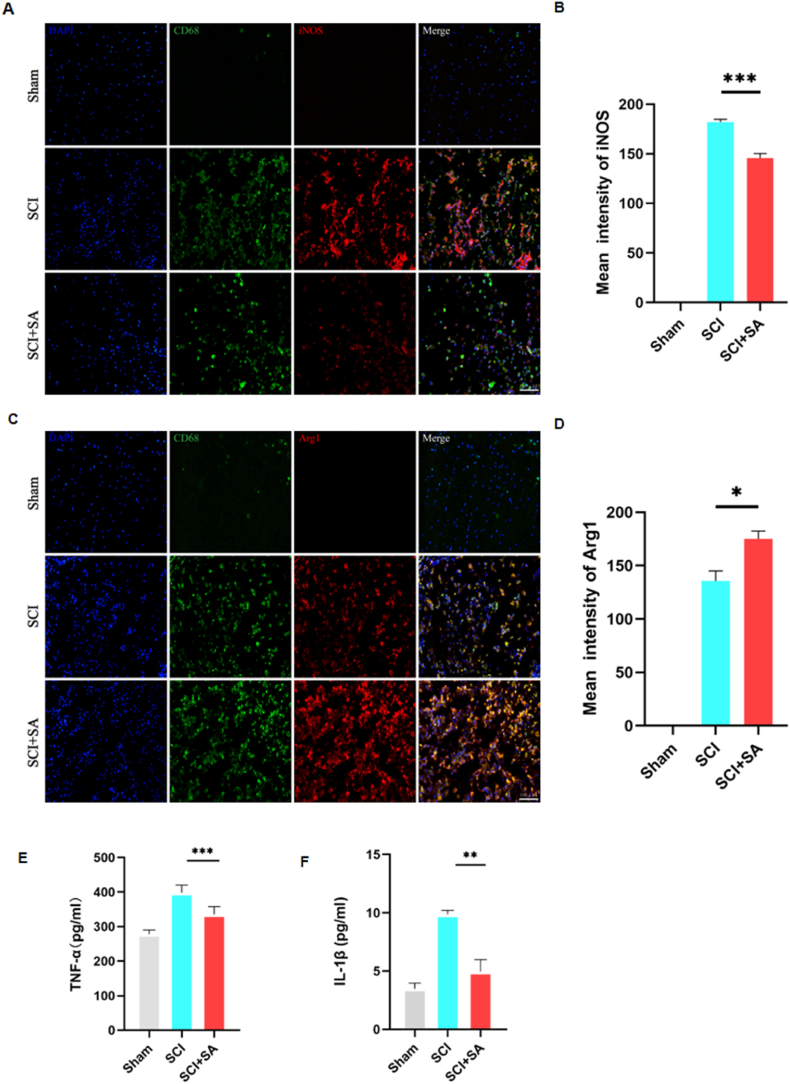
SA improved the recovery of SCI rats through the regulation of microglial polarization and inhibition of inflammatory response. **(A)** IF staining of iNOS/CD68 in spinal cord sections at day 3 post-SCI. **(B)** The image analysis results were presented as the relative mean intensity of the fluorescence of iNOS (n = 3). **(C)** IF staining of Arg1/CD68 in spinal cord sections at day 3 post-SCI. **(D)** The image analysis results were presented as the relative mean intensity of the fluorescence of Arg1 (n = 3). Data are shown as mean ± SEM, one-way ANOVA with Tukey's post hoc test, *P < 0.05, **P < 0.01, ***P < 0.001. **(E, F)** ELISA analysis was used to measure TNF-α and IL-1β contents in the injury epicenter of the spinal cord (n = 3 rats in each group).

To elucidate the neuroprotective efficacy of SA in SCI, we undertook an investigation focused on neuronal survival within the affected spinal cord areas. By employing NeuN, a specific neuronal marker, we observed a substantial increment in the survival rate of neurons in SA-treated rats on the third day following SCI, as measured against SCI rats without treatment (Fig. 7A and B). Furthermore, the Terminal deoxynucleotidyl transferase dUTP nick end labeling (TUNEL) assay revealed that while the quantity of apoptotic cells spiked subsequent to SCI, there was a discernible decrement in the TUNEL-positive cell population within SA-treated groups (Fig. 7C and D). Delving into the dynamics of macrophage polarization in the SCI context, we quantified the expression of iNOS and Arg1, markers indicative of the pro-inflammatory M1 and the anti-inflammatory M2 phenotypes, respectively. Data demonstrated an exacerbated presence of iNOS^+^CD68^+^ cells in unadulterated SCI rats compared to Sham controls; contrastingly, this M1-associated phenotype was mitigated with the administration of SA (Fig. 8A and B). Concurrently, the Arg1^+^CD68^+^ cell expression, reflective of the M2 phenotype, experienced upregulation in groups receiving SA intervention (Fig. 8C and D). To complement these findings, we conducted ELISA to ascertain the levels of pro-inflammatory cytokines TNF-α and IL-1β in the central injury zone of the spinal cord tissue. The secretion of both cytokines was found to be attenuated in the rats treated with SA (Fig. 8E and F). Taken together, these data lend strong evidence to the conclusion that SA not only fosters an environment conducive to neuronal survival but also attenuates the prolonged and aberrant activation of the immune microenvironment subsequent to SCI. By curbing the inflammatory response in affected rats, SA manifests a compelling therapeutic potential to promote neuronal resilience and ameliorate the adverse sequelae of spinal cord trauma.

### 3.6 SA reduced the activity of inflammatory factors in BV2 cells via inhibiting MAPKs/NF-kb signaling pathway

Microglia are the central nervous system's intrinsic macrophages and serve as the principal immunocompetent elements therein. Upon activation, they are capable of modulating the neuroenvironment through cytokine secretion [32]. Based on the findings of network pharmacology, we propose that SA can regulate inflammatory response after SCI through the MAPKs/NF-kB signaling pathway in microglia [[33], [34], [35]]. To test this hypothesis, we conducted an *in vitro* neuroinflammation analysis using microglia BV2 cells stimulated with LPS and INF-γ.

**Fig. 9:**
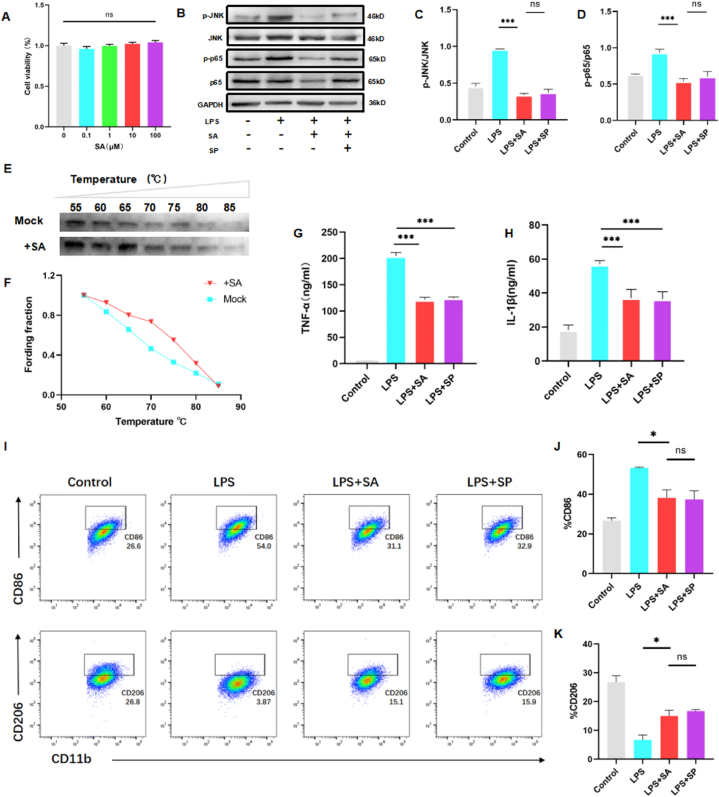
SA reduced the activity of inflammatory factors in BV2 cells via inhibiting MAPK/NF-kB signaling pathways. **(A)** BV2 cells were treated with 0–100 μM of SA for 24 h, and cell viability was determined by CCK-8. **(B)** Representative Western blot image of p-JNK, JNK, p-p65, and p65. **(C, D)** Analysis of p-JNK/JNK and p-p65/p65 ratio (data are shown as mean ± SEM, *P < 0.05, **P < 0.01, ***P < 0.001, n = 5). **(E)** Representative Western blot image of CETSA. **(F)** A marked shift of the melting curve of CETSA. **(G, H)** Levels of TNFα and IL-1β in culture supernatants were measured by ELISA (data are shown as mean ± SEM, *P < 0.05, **P < 0.01, ***P < 0.001, n = 3). **(I)** Flow Cytometry Analysis was used to measure the activation of BV2 cells by LPS-treated. CD86: an inflammatory marker; CD206: an anti-inflammatory marker. **(J, H)** Quantification of the percentage cell numbers of BV2 cells CD86^+^ or CD206^+^ expressing (data are shown as mean ± SEM, *P < 0.05, **P < 0.01, ***P < 0.001, n = 4).

In the wake of SA exposure, which was quantified via a CCK-8 assay, it was ascertained that concentrations of SA spanning the 0.1–100 μM range did not discernibly affect cellular viability (Fig. 9A). These findings permitted the selection of a 10 μM SA concentration for further *in vitro* exploration. Recognized literature implicates the MAPK (JNK) pathway in the inflammatory responses of macrophages and microglia [36,37]. An examination of protein levels by Western blot analysis, specifically phospho-JNK, total JNK, phospho-p65, and total p65, unveiled that SA treatment resulted in pronounced reductions in activated p-p65 and p-JNK in BV2 cells, indicative of MAPK (JNK) pathway inhibition following LPS + IFN-γ stimulation (Fig. 9B–D). The validity of these observations was juxtaposed against the established JNK inhibitor SP600125 (10 mM), which elicited commensurate effects [38,39]. Further extending these findings, SA was shown to quell the activity of NF-κB-regulated pro-inflammatory factors, such as IL-1β and TNF-α (Fig. 9G and H).

The binding affinity of SA to JNK was probed via a cellular thermal shift assay, which postulates that ligand interaction enhances the protein's thermal stability. Results corroborated that SA treatment indeed increased JNK's thermal stability (Fig. 9 E, F). Moreover, flow cytometry of BV2 cells, stimulated with LPS, reaffirmed SA's in vivo effect on microglial M1 polarization, underscoring a significant downregulation of CD86 (M1 marker), accompanied by a modest upturn in CD206 (M2 marker) expression (Fig. 9 I–K). In summation, our data converges to suggest that SA possesses the potential to bias macrophage polarization towards an M2 phenotype while concurrently repressing pro-inflammatory mediator activity via attenuation of the MAPKs/NF-κB signaling pathway.

## 4 Discussion

The pathological process after SCI can be categorized into primary and secondary damages. After the initial primary damage, secondary injury occurs due to factors such as hemorrhages, inflammation, oxidative stress, and apoptosis [5,38]. While inflammatory reactions can be beneficial in certain cases to prevent tissue damage, excessive inflammation can have a cytotoxic effect and worsen neuronal death [6,39]. As a result, numerous drug therapies have been tested to reduce neuroinflammatory responses, prevent the spread of secondary injury, and promote regeneration [40,41]. One such therapy involves the use of SA, a natural steroidal compound, which has been found to alleviate memory impairment and neuroinflammation by regulating the PAR-1 receptor [26]. The derivative, Sarsasapogenin-AA13, has also demonstrated the ability to inhibit inflammatory responses induced by LPS *in vitro* macrophage cells [42]. To further investigate this research and lay the foundation for future in-depth studies, we chose to employ a network pharmacology approach.

After searching the disease database, we discovered that there are 41 genes shared between the SA target genes and genes related to SCI. The results of the GO/KEGG enrichment analysis revealed that SA achieves its anti-SCI effects by regulating inflammation-related signaling pathways, such as Toll-like receptor MAPK(JNK) and TNF signaling pathways. Previous studies have shown that suppressing the expression of inflammatory mediators through specific signaling pathways can enhance recovery after SCI [[43], [44], [45]]. Building on these findings, we investigated the therapeutic potential of SA on SCI. Based on these findings, we investigated the therapeutic potential of SA on SCI and found that it significantly improved motor functional outcomes. Furthermore, SA treatment reduced activation of macrophages/microglia and secretion of cytokines in vivo. Through PPI network analysis, we identified five proteins from key genes that are related to SA in the treatment of SCI. These proteins are MAPK8, MAPK14, RELA, JUN, and TNF. Additionally, the affinity values of molecular docking were calculated to be less than −5.0 kcal/mol, indicating that these five key proteins have a favorable binding affinity with the corresponding compounds in SCI.

This study supports previous research that has established a correlation between proteins and pathways in SCI. Specifically, the JUN gene has been found to be highly expressed in cell proliferation, apoptosis, and differentiation [46]. Additionally, JUN has been shown to have a pro-apoptotic function in neuronal cells during the repair of the central nervous system [47]. TNF is considered a crucial therapeutic target for improving function after SCI [26,48]. Evidence suggests that inhibiting the MAPK pathway may reduce inflammation and tissue injury in rats with SCI [45,49]. However, our previous research did not find a significant difference in the expression level of MAPK14 in each group of LPS-stimulated BV2 microglial cells.

Our molecular docking analysis identified several inflammation-related genes as direct targets of SA, including MAPK8, MAPK14, and RELA. Among these targets, SA exhibited a stronger affinity towards MAPK8 (JNK). Based on these findings, we selected MAPK8 as our primary target and validated its role in the JNK pathway through our experiments. Our results further showed that SA may inhibit P65 by suppressing the phosphorylation of JNK, ultimately leading to a reduction in cytokine secretion. These findings were supported by our *in vitro* experiments. Numerous reports suggest that both MAPK and NF-kB signaling pathways play a role in regulating the immune microenvironment [50,51]. This study indicates that SA could inhibit JNK phosphorylation and suppress p65 activation, thereby potentially reducing neuroinflammation and promoting neuronal survival. These pathways may contribute to improved functional recovery after SCI. However, this study has limitations, including the use of small animal models, potential bias in the network pharmacology analysis, and the need for further investigation of SA's long-term effects and potential side effects. In addition, our current study also lacks detailed analyses of SA's ability to cross the BSCB, with existing research primarily outlining its systemic effects and central nervous system-wide impact. To address this deficit, we plan to rigorously explore SA's pharmacokinetics and its precise interactive dynamics at the BSCB. Upcoming work will involve both *in vitro* and in vivo explorations to establish the drug's concentration at the targeted spinal tissues, which will guide the calibration of *in vitro* models to closely replicate in vivo environments. Moreover, these endeavors will employ advanced models, such as primary cell cultures and microphysiological systems, and integrate in vivo pharmacodynamic data to ensure a more accurate representation of the SA's biological actions within spinal cord contexts.

## 5 Conclusion

In summary, This paper explores the therapeutic effects and mechanisms of SA action against SCI, using a combination of network pharmacology analysis and experimental validation. The findings contribute to a better understanding of the natural compounds found in Traditional Chinese Medicine therapies for treating SCI. However, it is important to acknowledge the limitations of this study. Firstly, there is a need for additional experimental validation to support our findings. Secondly, the databases used may not include all relevant compounds, targets, and pathways. Lastly, the mechanism underlying our results requires further exploration.

## Ethics statement

The Ethical Committee of Tianjin Medical University General Hospital approved all rat experiments (Tianjin, China, IRB2023-WZ-030).

## Data availability statement

All data generated or analyzed during this study are included in this article.

## CRediT authorship contribution statement

**Bing Fang:** Writing – original draft. **Liyue Wang:** Writing – original draft. **Song Liu:** Data curation. **Mi Zhou:** Data curation. **Hongpeng Ma:** Formal analysis. **Nianwei Chang:** Writing – review & editing. **Guangzhi Ning:** Writing – review & editing.

## Declaration of competing interest

The authors declare that they have no known competing financial interests or personal relationships that could have appeared to influence the work reported in this paper.
