# Supplementary material for: Sarsasapogenin regulates the immune microenvironment through MAPK/NF-kB signaling pathway and promotes functional recovery after spinal cord injury
Source: Heliyon. 2024 Jan 26;10(3):e25145. doi: 10.1016/j.heliyon.2024.e25145 (PMC10844052; doi:10.1016/j.heliyon.2024.e25145)
Supplement: Multimedia component 1 [file mmc1.docx]

**Supplementary Materials**

**For**

**Sarsasapogenin regulates the immune microenvironment through MAPK / NF-kB signaling pathway and promotes functional recovery after spinal cord injury**

Bing Fang^a,b,1^, Liyue Wang^a,1^, Song Liu^a^, Mi Zhou^a^, Hongpeng Ma^a^, Nianwei Chang^c,*^, Guangzhi Ning^a,*^

*^a^ International Science and Technology Cooperation Base of Spinal Cord Injury, Tianjin Key Laboratory of Spine and Spinal Cord Injury, Department of Othopaedics, Tianjin Medical University General Hospital, Tianjin, China*

*^b^ Department of Othopaedics, Affiliated hospital of Qingdao Binhai University, Qingdao, China*

*^c^ Tianjin University of Traditional Chinese Medicine, Tianjin, China*

^1^ These authors have contributed equally to this work.

***Correspondence to:** Guangzhi Ning, E-mail: gzning@tmu.edu.cn;

Nianwei Chang, E-mail: changnw@tjutcm.edu.cn

**Figure Legend for Supplementary Data**

**Supplementary Figure S1** corresponds to Figure 9(B) uncropped.


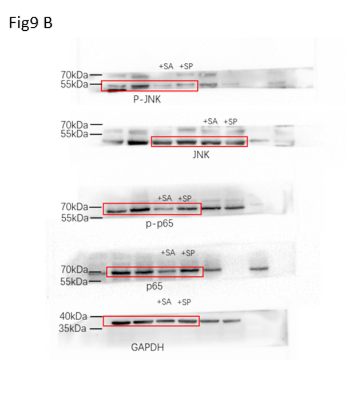


**Supplementary Figure S2** corresponds to Figure 9(E) uncropped.

**
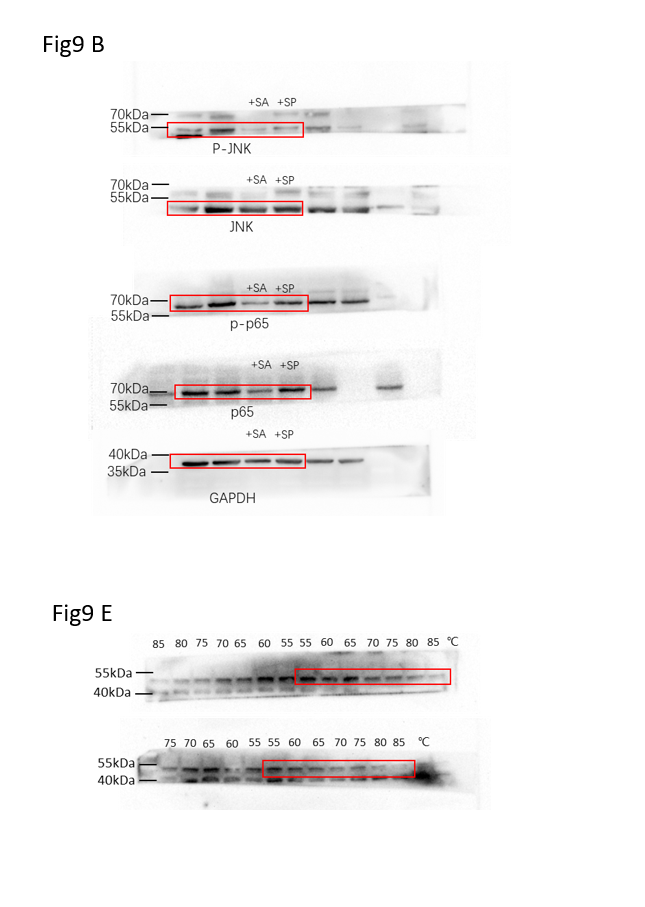
**
